# Balancing spontaneous and controlled thought: mind wandering, mindfulness, playfulness, and creativity in adolescence

**DOI:** 10.3389/fpsyg.2026.1706289

**Published:** 2026-10-12

**Authors:** David D. Preiss, Valeska Grau, David Torres Irribarra, Benjamín Carmona, Catalina Reszczynski, Juliana Oyarzún

**Affiliations:** 1 Pontificia Universidad Católica de Chile, Escuela de Psicología, Santiago, Chile; 2 Millennium Institute for the Study of Transformative Literacy and Learning (MISTRALL), Santiago, Chile; 3 Center for the Development of Inclusion Technologies-CEDETI UC, Santiago, Chile

**Keywords:** convergent thinking, creativity, divergent thinking, mind wandering, mindfulness, playfulness

## Abstract

This study investigated how daydreaming frequency, the emotional valence of mind wandering, mindfulness, and multiple facets of playfulness predict creativity across verbal, figural, and mathematical domains in a sample of Chilean high school students (*N* = 405). Participants completed self-report measures of attentional dispositions and playfulness, along with five creativity assessments targeting verbal and mathematical divergent thinking, verbal and figural remote associative processing, and figural product composition. A structural equation model controlling for gender and school revealed that daydreaming frequency was the trait most consistently related to creativity. Across four of the five measures—including verbal and figural remote associations, figural product composition, and mathematical divergent thinking—higher daydreaming frequency was associated with better performance, supporting the notion that spontaneous thought facilitates creative performance across several domains. In contrast, affectively valenced mind wandering showed negative associations with creativity: positive mind wandering was negatively related to both verbal creative tasks, and negative mind wandering was negatively associated with verbal divergent thinking. Mindfulness was positively associated with creative performance only in the figural product composition task, which required evaluating and integrating ideas. A statistically significant moderation effect was observed: mindfulness was positively associated with performance in this task when daydreaming was low, but at higher levels of daydreaming, the association reversed. Playfulness showed heterogeneous effects: intellectual playfulness was positively associated with verbal divergent thinking, whereas general and lighthearted playfulness were negatively associated with verbal and mathematical divergent thinking, respectively. Other-directed playfulness was positively linked to figural remote association performance. These results suggest that a disposition to mind wander may serve as a developmental scaffold for creativity and facilitate access to distant associations in tasks that require idea generation or remote association. A disposition toward mindful awareness may be beneficial for complex creative tasks. Future research on playfulness and creativity should focus on how intentionality and cognitive guidance shape creative processes, given that these qualities are more closely associated with intellectual playfulness. Implications for educational practice underscore the value of balancing opportunities for spontaneous thought with the development of mindful awareness during creative work.

## Introduction

Over the past decade, interest in the relationships among mind wandering, mindfulness, and creativity has increased significantly. While some studies have investigated how state-level mind wandering or state-level mindfulness relate to creativity (Murray et al., 2021; Ostafin and Kassman, 2012; Smeekens and Kane, 2016), others have focused on trait-level associations (Agnoli et al., 2018; Preiss et al., 2016; Zedelius and Schooler, 2015). This body of research has improved our understanding of these psychological constructs, examined their impact on the creative process, and helped close the gap between trait-level and state-level processes. Building on this foundation, this study explores which emotional contents of trait mind wandering, in addition to daydreaming frequency, are most strongly linked to creativity, and how mind wandering and mindfulness together influence creative behavior. In doing so, this study aims to advance the integration of the overlapping fields of mindfulness and mind wandering (Mrazek et al., 2014a; Mrazek et al., 2014b; Preiss and Carmona, 2023; Zedelius and Schooler, 2015). Additionally, to expand on previous research examining the connections between traits and creative performance, the study explores the role of playfulness as a relevant personality trait (Proyer et al., 2019).

Today, there is an increasing interest in understanding how attentional dispositions and other traits relate to creativity. The mechanisms underlying the influence of these traits are unclear, and several hypotheses are currently being considered (Rodriguez-Boerwinkle et al., 2025). We still need to understand whether mind wandering and mindfulness affect creative thinking in different, perhaps opposing, ways (Zedelius and Schooler, 2015), or whether they serve complementary roles at different stages of the creative process (Preiss, 2022b; Preiss and Carmona, 2023). Furthermore, mind wandering involves different types of content (Seli et al., 2015; Torres-Irribarra et al., 2019; Zedelius et al., 2021) and mindfulness has distinct facets (Baer et al., 2006, 2008), which may have varying links to creative performance depending on the task involved. We also need to understand how these attentional dispositions relate to creativity when other traits, such as playfulness, are taken into consideration.

Along with contributing to research on the links between traits and creativity, this study aims to fill a gap in the literature by exploring how mind wandering, mindfulness, and playfulness collectively relate to creative performance in adolescents—a group that has been overlooked in previous research, as most studies in this field have focused on undergraduate students or adults. While earlier studies have primarily examined these traits in adults and college students, little is known about their impact on creative thinking during a developmental stage in which attentional control, emotional regulation, and cognitive flexibility are still emerging. By investigating multiple creativity domains, distinguishing the emotional content of mind wandering, and considering specific facets of playfulness, the study provides a deeper understanding of how these traits influence different aspects of creativity. This multidimensional approach not only enhances theories connecting spontaneous and controlled thinking to creativity (Preiss et al., 2020; Zedelius and Schooler, 2015) but also offers practical insights for educational environments aiming to nurture creative potential during a crucial stage of development.

### Studying and measuring creativity

According to the field’s standard definition, creativity is the capacity to produce work that is both novel and appropriate (Runco and Jaeger, 2012). Creativity demands novelty, and novelty requires originality. Yet it is not sufficient. Creative products must also satisfy a criterion of fit, usefulness, or appropriateness. As noted by Runco and Jaeger (2012), the requirement of appropriateness was established very early in the field, as researchers such as Barron, Guilford, and Stein proposed a bipartite definition of creativity in the 1950s. This definition has informed creativity assessment, with measures of the construct involving at least novelty and appropriateness components.

A classic approach to creativity, known as the four Ps, considers the following dimensions: person, process, product, and press (Rhodes, 1961). The person dimension focuses on the individual traits, cognitive abilities, personality, and motivation influencing creative potential. The process dimension examines the mental operations and strategies involved in creative thinking, including divergent and convergent thinking, problem-solving, and idea generation and evaluation. Much of creativity research has focused on divergent thinking (Reiter-Palmon et al., 2019), but it is not the only process engaged in creative behavior. The product dimension refers to the outcomes of creative activity, typically evaluated based on originality and usefulness. Finally, the press dimension highlights the role of environmental factors that can either foster or inhibit creativity. More recently, Kaufman and Beghetto (2009) introduced a developmental model of creativity comprising four levels: mini-c, little-c, Pro-c, and Big-C. Mini-c refers to personally meaningful creative insights; little-c creativity is reflected in everyday creative expressions; Pro-c represents creativity within a professional domain; Big-C denotes eminent creativity. Although the model suggests a developmental trajectory, the authors emphasize that progressing through each stage is not a prerequisite for achieving eminent creativity.

This study focused on the person and process components and the little-c level of creativity. Specifically, the current study examines how individual traits relate to processes such as divergent and convergent thinking, as well as to creative products. Since creativity is domain-specific (Hennessey and Amabile, 2010), we employed a diverse set of instruments that assess creativity across verbal, figural, and mathematical domains. These measures were selected to reflect the cognitive processes discussed in the literature on mind wandering, mindfulness, and playfulness—especially the roles of divergent thinking, convergent thinking, and remote associative processing, which are central to creative thinking.

### Mind wandering and creativity

Mind wandering is a type of cognition characterized by self-generated thoughts, unrelated to the current task, that occur independently of external stimuli (Preiss, 2022a; Smallwood and Schooler, 2015). In his influential work on daydreaming, Singer (1975) distinguished between constructive daydreaming, dysphoric daydreaming, and poor attentional control. The type of mind wandering most closely linked to creativity is constructive daydreaming, defined as “playful, wishful imagery, and planful, creative thought” (McMillan et al., 2013, p. 1). The connection between the other two most negative forms of mind wandering and creativity has been discussed less, so exploring the relationships between different affective types of mind wandering and creativity might help better understand the mind wandering— creativity link.

The evidence on the link between mind wandering and creativity is mixed. In an influential study, Baird et al. (2012) evaluated participants’ improvement on a divergent thinking task after an incubation period with different levels of cognitive demand. Participants who engaged in a low-demand task during incubation showed significantly greater gains in their pre-to-post divergent thinking scores than in the other conditions (high-demand task, no task, and no incubation). Because those in the low-demand condition also reported a markedly higher frequency of task-unrelated thoughts during incubation than in the other conditions, this study supported the idea that mind wandering is connected to divergent thinking. However, some studies have been unable to replicate the positive link between wandering and creativity suggested by this research. For instance, two experiments found that the frequency of mind wandering during incubation does not predict post-incubation performance on divergent thinking tasks (Murray et al., 2021; Smeekens and Kane, 2016). Likewise, another study observed no significant differences in divergent thinking performance across three incubation conditions—uninterrupted, trivia questions, or mind wandering probes—and found that all conditions performed similarly to a no-incubation control group (Steindorf et al., 2021).

Beyond the study of the impact of mind wandering during incubation, researchers have reported positive links between mind wandering measures and different types of creative tasks, such as insight problems (Tan et al., 2015), the compound remote associations task (Leszczynski et al., 2017; Preiss et al., 2016), and a divergent thinking test (Preiss et al., 2016). Still, other studies found mixed results. For example, a study that included both mindfulness facets and mind wandering types found, using path analysis, that originality was negatively affected by spontaneous mind wandering and positively influenced by deliberate mind wandering (Agnoli et al., 2018). Also, laboratory findings have not always been consistent with real-world evidence or across different measures of creativity. A study found a positive association between laboratory measures of mind wandering (task-unrelated thought probes collected during tasks) and highly creative scores in divergent thinking, but not with overall creative thinking, after accounting for variation in executive control abilities. Daily-life measures of mind wandering (task-unrelated thought probes collected via experience sampling) were no better predictors of creative thinking than laboratory measures (Rodriguez-Boerwinkle et al., 2025). Finally, other studies have not replicated the mind wandering—creativity link. One study reported that participants with high levels of mind wandering performed worse on a divergent thinking task than those with low levels (Hao et al., 2015). Another study, focusing on attention control, reported a non-significant negative correlation between the proportion of task-unrelated thoughts and creativity on a divergent thinking task (Frith et al., 2021).

The differences between studies may arise from how mind wandering is understood and how creativity is assessed. Looking not just at how often daydreaming happens but also at what people think about during those times—namely, the content of mind wandering—could provide a deeper insight into how it affects creative thinking (Murray et al., 2021). One important but less-studied aspect of mind wandering related to creativity is its emotional content. Mind wandering can vary significantly in emotional tone, and positive feelings during these episodes might enhance creative thinking, as positive emotions are associated with creativity (Baas, 2019; Hennessey and Amabile, 2010). For example, mind wandering tends to be associated with positive mood when the content is interesting (Baas, 2019; Baas et al., 2008; Franklin et al., 2013). Since evidence indicates that a positive mood enhances cognitive flexibility and, in turn, increases creativity, it is reasonable to hypothesize that mind wandering in a positive mood could also support creativity.

Rodriguez-Boerwinkle et al. (2025) propose seven hypotheses about the mind wandering—creativity link. Together, these hypotheses reflect different possible mechanisms linking spontaneous thought to creative performance. Four of these hypotheses are directly relevant to the present study’s design, so we will reference them here. First, their suggestion that mind wandering may serve as a developmental scaffold resonates with our adolescent sample, in which attentional control and cognitive flexibility are still emerging. By examining divergent and convergent thinking in high school students, we can test whether spontaneous thought provides early opportunities for idea exploration during this developmental stage. Second, the hypothesis that mind wandering facilitates creativity after an impasse aligns with our interest in exploring whether mind wandering is positively associated with remote associative processing measures, which often require incubation and benefit from mental breaks that allow unconscious reorganization (Wu et al., 2020). Third, the view that spontaneous thought enhances originality by bringing remote associations to mind also relates to this same interest. Finally, the hypothesis that meta-awareness moderates the effects of mind wandering dovetails with our inclusion of mindfulness measures, as attentional regulation and present-moment awareness may influence whether mind wandering supports or undermines creative outcomes.

### Mindfulness and creativity

Mindfulness refers to the moment-to-moment awareness of one’s experience. The concept considers contemplative practices aimed at enhancing well-being (Dahl and Davidson, 2019) and cognitive training strategies designed to improve sustained attention and executive functioning (Chiesa et al., 2011; Schooler et al., 2014). Three main aspects of mindfulness have been identified in the literature: attentional control, emotional regulation, and self-awareness (Tang et al., 2015). Mind wandering and mindfulness can be viewed as opposites due to their contrasting attentional orientations (Mrazek et al., 2012; Mrazek et al., 2014a; Mrazek et al., 2014b; Schooler et al., 2014; Zedelius and Schooler, 2015). Mind wandering involves stimulus-independent thoughts (Smallwood and Schooler, 2015), whereas mindfulness entails active engagement with present-moment experience (Dahl and Davidson, 2019). In addition to their opposite attentional orientations, mind wandering has been linked to poor executive control (Kane and McVay, 2012) and emotional dysregulation (Deng et al., 2014), while mindfulness is associated with enhanced executive functioning (Cásedas et al., 2020) and mental health (Creswell, 2017). Despite these differences, certain forms of mind wandering may relate to aspects of mindfulness. For instance, mind wandering with positive emotional valence has been found to correlate positively with all facets of mindfulness as measured by the *Five Facet Mindfulness Questionnaire* (hereafter, FFMQ). In contrast, mind wandering with negative emotional content is negatively associated with all these facets but the observing facet (Torres-Irribarra et al., 2019).

Research from training and intervention studies suggests that mindfulness can enhance creativity, but the relationship between mindfulness and creativity is complex and influenced by several additional factors. Ostafin and Kassman (2012) showed that both trait mindfulness and mindfulness training are associated with better insight, but not with non-insight problem-solving. Greenberg et al. (2012) reported that mindfulness training can reduce cognitive rigidity, a primary impediment to creative problem-solving. By fostering cognitive flexibility, mindfulness may enable individuals to transcend their established mental frameworks and generate innovative solutions. Hughes et al. (2023) conducted a meta-analysis of mindfulness interventions and found significant effects on creative performance for both control group designs and pretest–posttest designs. These effects were moderated by factors such as task type, control group characteristics, and intervention length. As regards specific interventions for school-age children, there is mixed evidence. A mindfulness intervention involving 150 schoolchildren (mean age 10) showed positive effects on creativity (Doron, 2016). In contrast, another study involving 81 students (mean age 16) found no impact on creativity but did observe positive effects on mental health and emotion regulation (Luong et al., 2019).

Trait studies confirm the complexity of the mindfulness—creativity link and the challenges of studying the simultaneous impacts of mind wandering and mindfulness on creativity. A meta-analysis by Lebuda et al. (2016) found a relatively modest correlation (r = 0.22) between trait mindfulness and creativity. Notably, the association was weaker for the awareness dimension of mindfulness than for the open-monitoring dimension. Researchers have examined which aspects of mindfulness as a trait are more closely associated with creativity, using instruments such as the FFMQ (Baer et al., 2006). In a study with undergraduates, Agnoli et al. (2018) reported that the Non-reactivity facet was negatively associated with originality in a divergent thinking task, whereas the Describing facet was positively associated with creative achievement on a self-report measure. The Describing facet of mindfulness predicted creative achievement only at low and medium levels of deliberate mind wandering. In the divergent thinking task, at low levels of spontaneous mind wandering, increases in the Describing facet were associated with a significant decrease in response originality. The Awareness facet predicted higher originality scores at high levels of deliberate mind wandering. Across four studies with undergraduates, Baas et al. (2014) found that only the capacity to observe and attend to stimuli consistently predicted creativity. Specifically, individual differences in the Observation facet, as well as improvements in observation skills resulting from mindfulness training, were positively associated with creativity measures. Zedelius and Schooler (2015) also examined the influence of mind wandering and mindfulness on creativity. Using the Mindful Attention Awareness Scale (MAAS; Brown and Ryan, 2003), the researchers framed mind wandering and mindfulness as opposite ends of a continuum. Higher levels of mindfulness were generally associated with reduced performance on creative problem-solving tasks. However, greater mindfulness was positively associated with problem-solving success when participants employed an analytic strategy, whether spontaneously or when instructed to use it. Conversely, mindfulness was negatively associated with performance when participants spontaneously relied on an insight-based strategy, and showed no significant effect when insight was explicitly instructed.

### Playfulness and creativity

Playfulness is a multidimensional personality trait that includes cognitive spontaneity, social engagement, and a joyful approach to everyday activities (Barnett, 1990). It has been defined as “an individual differences variable that allows people to frame or reframe everyday situations in a way that they experience them as entertaining, and/or intellectually stimulating, and/or personally interesting” (Proyer, 2017, p. 114). Although playfulness has been associated with life satisfaction (Proyer and Tandler, 2020) and various personality traits (Barnett, 2007; Proyer, 2012), its relationship with creativity remains less explored. Proyer et al. (2019) suggest that playfulness, as a trait linked to positive affect, may facilitate creative processes. From this perspective, playfulness may enhance creativity by fostering emotional openness, cognitive flexibility, and a willingness to engage with novelty. In line with this view, Preiss (2022b) has proposed that creative individuals often exhibit flexible cognitive control, enabling them to shift between mind wandering and metacognitive processes in response to task demands. Playfulness may support this flexibility by encouraging a balance between free, unconstrained ideation and focused, goal-directed processing. Such cognitive versatility promotes the exploration of novel associations and perspectives, thereby creating conditions conducive to creative thought (Zabelina and Robinson, 2010).

Despite this promising theoretical foundation, empirical research on the relationship between playfulness and creativity in school-age children remains limited. To date, only a few studies have examined playfulness as a trait variable in relation to creativity (Bateson and Nettle, 2014). Proyer (2012) assessed the relationship between playfulness and divergent thinking in undergraduate students using the Adult Playfulness Scale, which comprises five dimensions: Spontaneous, Expressive, Fun, Creative, and Silly. Of these, only creative and spontaneous playfulness showed significant correlations with total originality and fluency scores in the subtests of the “ingenuity” factor of the Berlin Intelligence Structure Test.

The four-facet framework of playfulness proposed by Proyer (2017), which includes Other-directed, Lighthearted, Intellectual, and Whimsical playfulness, offers a promising approach to understanding how playfulness contributes to creative performance. According to Proyer (2017), the Other-directed dimension reflects enjoying playing with others and using playfulness to enrich social interactions and ease tension, including friendly teasing. The Lighthearted dimension involves making deliberate space for play in one’s routine, including improvising and therefore not stressing too much about future consequences. The Intellectual dimension captures the enjoyment of playing with ideas, solving problems, exploring different solutions, and preferring complexity over simplicity. Finally, the Whimsical dimension reflects a fondness for odd or quirky situations, an enjoyment of unusual activities, and an ease in finding amusement in everyday interactions.

### Study rationale and hypotheses

Building on previous research and theoretical models, this study examines how attentional dispositions—specifically mind wandering and mindfulness—and the personality trait of playfulness relate to creativity across various domains. We hypothesize that mind wandering (daydreaming frequency), a form of spontaneous, self-generated thought, will be positively associated with creative performance, particularly on tasks requiring divergent thinking and associative processing. This is supported by evidence that constructive daydreaming aids ideation and insight. Similarly, given the role of positive affect in creativity, we expect positive mind wandering to be positively related to creativity, beyond daydreaming frequency. To contrast the impact of positive mind wandering, we will also investigate how negative mind wandering relates to creativity. Mindfulness is expected to positively influence creativity on tasks that involve creative evaluation, benefiting not only from reduced cognitive rigidity but also from greater emotional regulation and present-moment awareness. Finally, we explore the role of playfulness, understood as a multidimensional trait. Based on Proyer’s (2017) model, we hypothesize that intellectual playfulness will be positively associated with creativity, particularly in tasks involving idea generation and associative thinking. Conversely, we expect that lighthearted playfulness may be negatively associated with performance on more structured or analytical creative tasks. We do not expect to find associations between creativity and whimsical and other-directed playfulness. These hypotheses suggest that not all forms of playfulness promote creativity equally, and that their effects may differ depending on task requirements. To capture the association between these traits and various aspects of creativity, the present study examines divergent thinking, convergent thinking, and creative products across verbal, figural, and mathematical domains.

## Methods

### Sample

A total of 405 high school students from three schools in Santiago, Chile (referred to here as Schools A, B, and C) were recruited for the study. All three schools were in urban areas of the Metropolitan Region. In Chile, schools are classified by a categorical socio-economic group (SEG) level indicator based on the average socioeconomic characteristics of their students. This classification is created by the State’s Agencia de Calidad de la Educación (Agency for Educational Quality) using parent-reported data collected through the national standardized assessment (SIMCE), which includes parental education, household income, and related contextual indicators. In 2024, School A was rated with a medium-high SEG, School B with a medium-high SEG, and School C with a medium-low SEG. In the 2024 national standardized assessment (SIMCE), School A scored highest in Mathematics, followed by School C and School B. In Reading, School B scored slightly higher than School A, while School C scored lower than both. All three schools scored below the regional average in Mathematics, while Schools A and B scored above average in Reading, with School C below. At the time of the study, students were enrolled in either 11th or 12th grade. A detailed breakdown of participants by school and gender is shown in Table 1. Because the procedure involved multiple sessions (see the Procedure section for details), the number of responses varies across tests, since not all participants attended every session. The final sample size (*N*) for each analysis is listed in each relevant table.

**Table 1:** Number of participants by school and gender.

School	Gender	N
A	Male	126
	Female	110
	Non-binary	0
	Total	236
B	Male	14
	Female	15
	Non-binary	2
	Total	31
C	Male	121
	Female	15
	Non-binary	2
	Total	138
Total	Male	261
	Female	140	Non-binary	4
	Grand total		405

Four participants identified with a gender outside the binary categories of female and male. Because of the small size of this subgroup, it was not methodologically feasible to estimate the effects of this gender identity category with sufficient statistical reliability. Therefore, their gender data were treated as missing in the analysis.

### Measures

#### Mind wandering

Two instruments were used to assess mind wandering: the Chilean Spanish version of the *Daydreaming Frequency Inventory* (Daydreaming; Preiss et al., 2016) and the *Scale of Mind Wandering and Emotional Valence* (Torres-Irribarra et al., 2019). The *Daydreaming Frequency Inventory* is a 12-item multiple-choice scale, with each item scored on an ordinal scale from 1 to 5. Observed scores were calculated as the average of the 12 items, excluding participants with missing data. Internal consistency for the observed scores was high, with Cronbach’s alpha (*α*) and McDonald’s omega (*ω*) both at 0.93. The *Scale of Mind Wandering and Emotional Valence* has two subscales: (1) *Positive Mind Wandering* (Positive MW) and (2) *Negative Mind Wandering* (Negative MW). Each subscale includes 13 Likert-type items, with response options ranging from 1 (“Strongly disagree”) to 4 (“Strongly agree”). In each scale, observed scores are calculated as the mean of the 13 items. Reliability estimates were high for both subscales, with Cronbach’s alpha (α) and McDonald’s omega (ω) values of 0.91 for the Positive MW subscale and 0.95 for the Negative MW subscale.

#### Mindfulness

The Chilean adaptation (García-Rubio et al., 2019) of the *Child and Adolescent Mindfulness Measure* (CAMM; Greco et al., 2011) was used to assess mindfulness. The CAMM assesses tendencies toward reduced present-moment awareness and judgmental reactions to thinking and feeling. The instrument features a single scale with seven Likert-type items, with response options ranging from 1 (“Never or very rarely true”) to 5 (“Very often or always true”). All items are negatively worded (e.g., “It is hard for me to pay attention to just one thing at a time”) and were reverse-coded before analysis to improve interpretability. Internal consistency for the observed scores (i.e., the mean of the seven items, using listwise deletion) was high, with Cronbach’s alpha (α) and McDonald’s omega (ω) both equal to 0.88.

#### Playfulness

Trait playfulness was measured using the *OLIW-Youth questionnaire* (Proyer and Tandler, 2020). This instrument has four subscales, each containing a different number of Likert-type items rated from 1 (“Strongly disagree”) to 7 (“Strongly agree”). These subscales represent distinct dimensions of the playfulness personality trait—Other-directed, Lighthearted, Intellectual, and Whimsical—as defined by Proyer (2017). A fifth subscale is a 5-item general index of playfulness, integrated into the OLIW framework and originally named *the Short Measure of Adult Playfulness* (Proyer and Tandler, 2020). Both the 18-item OLIW-Youth questionnaire and the 5-item Short Measure of Adult Playfulness, which were provided by the original authors, were translated from German into Spanish and then refined through cognitive interviews (Willis, 2005) to ensure linguistic and conceptual equivalence. Reliability coefficients (Cronbach’s alpha and McDonald’s omega) for each subscale are reported in Table 2.

**Table 2:** Reliability measures of the OLIW sub-scales.

Sub-scale	Cronbach’s alpha	McDonald’s omega
Other-directed	0.75	0.75
Lighthearted	0.74	0.74
Intellectual	0.75	0.75
Whimsical	0.82	0.83
General Playfulness	0.77	0.80

#### Creativity

Five instruments were used to assess different dimensions and processes of creativity.

*Alternate Uses Test (AUT)*. The AUT is a classic test of divergent thinking (Guilford, 1967). It asks participants to generate as many alternate uses as possible for a common object within a limited time frame. In this study, two items were used: a newspaper sheet and a metallic spoon. Each item was presented with a three-minute time limit. Three indicators were derived from participants’ responses.

1) Fluency was defined as the number of responses that met two criteria: (a) plausibility (e.g., using a newspaper as a towel was considered plausible, whereas using it to fly was not), and (b) novelty of use (i.e., conventional uses such as “reading” were excluded, as per task instructions).
2) Flexibility was defined as the number of distinct categories in a participant’s set of responses. Categories were developed by experienced AUT raters after reviewing the entire sample, following one of the procedures described by Reiter-Palmon et al. (2019). To minimize overlap with fluency, categories were defined broadly. Five categories were identified for the *newspaper* (Cleaning, Arts and Crafts, Fire-making, Wrapping/Protection, and Accessories/Clothes), and six for the *spoon* (Cutting/Carving, Container/Holding, Playing, Harming, Arts and Crafts, and Makeup Tool). An additional “Other” category was used for responses not fitting any predefined category. In rare cases in which multiple responses were classified as “Other,” conceptually distinct responses were treated as separate categories at the rater’s discretion.
3) Originality was rated on a 4-point ordinal scale (1 = least original, 4 = most original), based on two dimensions: the response’s frequency within the sample and its perceived ordinariness in everyday life, as judged by independent raters. The final originality score for each participant was the mean of their two highest-rated responses, following a method previously recommended (Smeekens and Kane, 2016). This approach minimizes reliance on the quantity of responses and reduces the influence of outlier responses.

Scoring was carried out by two trained judges following a three-step process:

1) Step 1: Shared Scoring. Judges collaboratively rated a subset of responses to develop consistent criteria.
2) Step 2: Calibration. Judges independently rated a portion of responses (about 20% for fluency and 30% for originality and flexibility), then conducted a reliability check, resolved discrepancies, and refined scoring criteria.
3) Step 3: Individual Scoring. Judges independently rated the remaining responses, with roughly 5% of the sample double-scored to assess inter-rater reliability. Judges were blind to which responses were double-coded.

This procedure was applied separately for each item (newspaper and spoon) and for all indicators. Three reliability metrics, reported in Table 3, were used to assess scoring consistency: (a) raw agreement (defined as a score difference of ≤1 for fluency and flexibility; ≤0.5 for originality), (b) Pearson’s correlation between judges’ scores, and (c) Quadratic Weighted Kappa (Fleiss and Cohen, 1973). Final scores for the descriptive analyses were calculated as the mean of both items, while in the structural equation models, the final score was calculated using all indicators as a single latent variable named “Verbal Divergent Thinking.”

**Table 3:** Inter-rater reliability measures for the AUT scores.

Score	% Raw Agreement	Pearson’s Cor.	Weighted Kappa	N
Newspaper Fluency	90%	0.96	0.96	90
Spoon Fluency	93.2%	0.97	0.97	73
Newspaper Flexibility	86.7%	0.75	0.74	120
Spoon Flexibility	90.8%	0.93	0.93	120
Newspaper Originality	89.9%	0.86	0.85	119
Spoon Originality	89.7%	0.87	0.87	117

N is the total amount of double scored participants through all the scoring steps.

*Verbal Remote Associations Task (VRA-T)*. We used a revised Chilean test of Verbal Remote Associations (Mourgues et al., 2014). The original instrument was updated to better meet today’s students’ needs. The revision process was validated through cognitive interviews that examined participants’ understanding of the items, the clarity and familiarity of the terminology, and the thought processes involved in completing the task, using the think-aloud method (Padilla and Leighton, 2017). These interviews were part of a pilot study with 12 students in 7th, 8th, and 11th grade. The main changes involved replacing outdated content with new items that better reflect modern language use. For example, one item previously used the term *‘bota de vino’* (*‘wineskin’* in English), a term now less familiar to younger generations. Out of the 14 original items, two were removed, and nine were replaced with new ones. No modifications were made to the instructions or the overall structure of the original test. The final task consisted of 12 items. In each item, participants were presented with three cue words and asked to identify a fourth, target word. The target word was related to the cue words such that one cue word was a synonym of the target, a second formed a common phrase when combined with the target, and a third formed a compound word with the target. Participants had eight minutes to complete the task. The score was based on the total number of correct responses. Reliability was high, with *α* = 0.90 and *ω* = 0.91. Final scores for the descriptive analyses were obtained by summing the correct responses.

*Figural Remote Associations Task (FRA-T).* We designed a new assessment tool to measure remote associative thinking independently of verbal skills. Participants were shown three images representing a hidden object in different ways: one resembles the shape, another is related by function, and the third is associated by context but not directly connected. The test consisted of three steps: (1) an explanation of the rules and two sample items, during which the examiner solves and describes each; (2) two practice items, with participants given two minutes to respond, followed by the examiner providing the correct answers and explanations; and (3) the actual test, which includes 13 items that participants have 10 min to complete in any order. A brief overview of the test specifics is available in the notes of Figure 1. The test was administered on paper, with all images printed in full color, as illustrated in Figure 1. We believe this design enables semantic processing through visual stimuli. The observed score was the total number of correct responses. Reliability was high, with α = 0.91 and ω = 0.91. Final scores for the descriptive analyses were calculated as the sum of correct responses.

**Figure 1:**
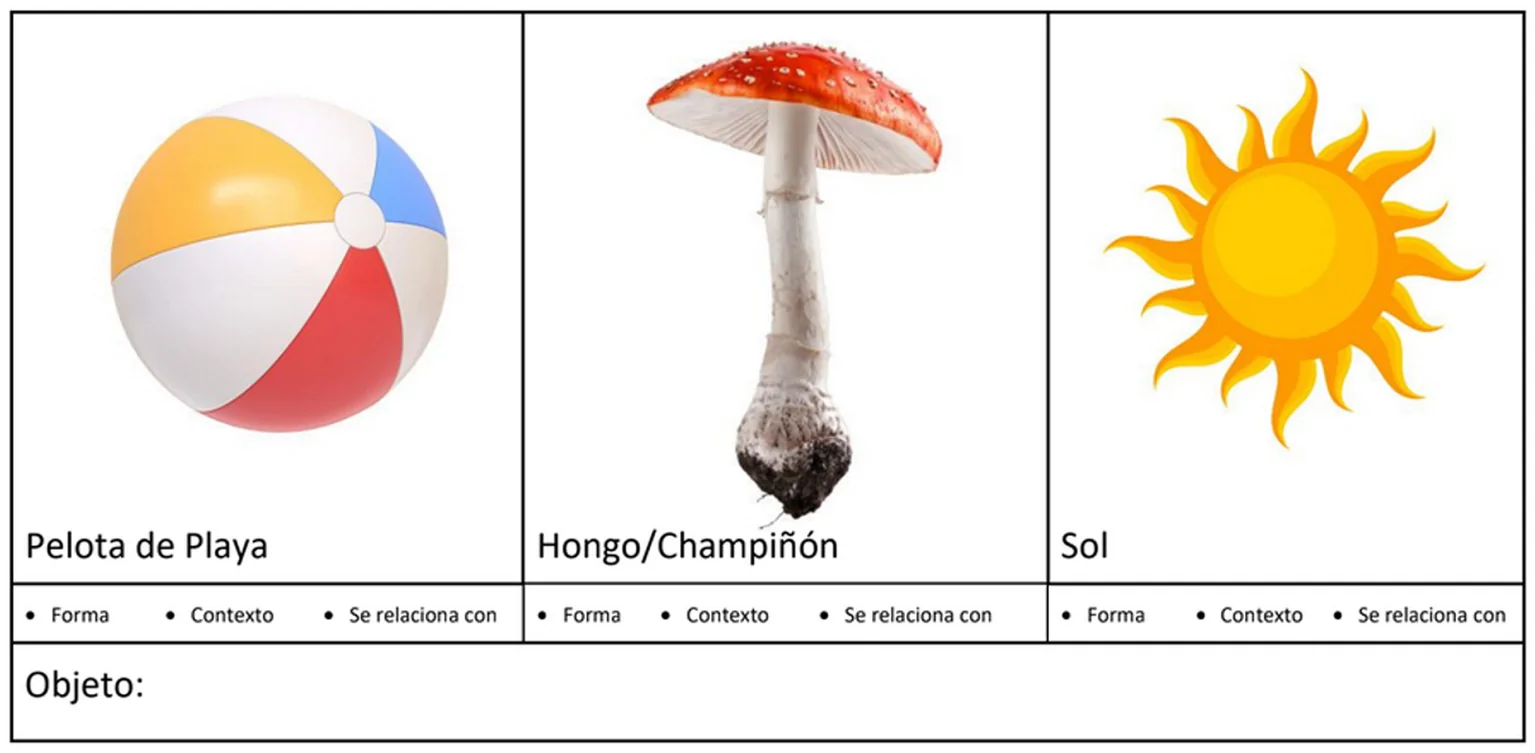
Example item from the third section of the FRA-T. Note. From left to right, the images are labeled in Spanish as: (1) “Pelota de playa (Beach ball), (2) Hongo/Champiñón (two common Chilean names for Mushroom), and (3) Sol (Sun). Participants write their response in the lower section labeled Objeto (Object). In this example, the response is Quitasol (Parasol in English). The three options displayed beneath each image (Shape, Context, and Related to) represent the possible types of associations between the image and the target object. Participants are asked to indicate the association that best applies to each image. In this example, the correct associations, from left to right, are: Context, because beach balls are commonly used at the beach; Shape, because mushrooms and sunshades share a similar shape; and Related to, because the function of a sunshade is to provide protection from the sun.

*Mathematical Fluency Test (MFT).* The MFT was developed to assess divergent thinking in mathematics and it is inspired by a task from the Creative Ability in Mathematics Test (CAMT; Balka; Erbas and Bas, 2015). The original CAMT presents participants with a scenario—such as an airline considering the purchase of passenger airplanes—and relevant mathematical data, including costs, projected profits, and other details. Participants are asked to generate as many mathematical problems as possible based on the provided information. For the MFT, the scenario was adapted to feature a fictional social media influencer creating YouTube and TikTok videos. The data included video durations, production costs, earnings per view, and additional details. This modification was intended to make the task more engaging and relatable for adolescent students. Participants were instructed to generate as many math problems as possible without providing solutions. For instance, from the data “For every 20 people who view his TikToks, Pablo [the influencer] earns 10 Chilean pesos,” a sample problem could be: “How many views does Pablo need on TikTok to earn 10,000 Chilean pesos?” Participants had 8 min to complete the task. The final score was based on the number of solvable math problems that incorporated at least one of the provided data points. Scoring was similar to the procedure used for the AUT. This method was deemed appropriate because the MFT, although not an AUT, is a divergent thinking test that yields a count variable. Among 376 participants, 133 were double scored, resulting in a raw agreement of 91.7% (with a ± 1 score difference), a Pearson’s correlation of 0.94, and a weighted kappa of 0.94.

*Figures and Colors Test (FCT)*. The FCT assessed figural creativity through a digital drawing activity. Participants had a total of 30 min—10 min for practice and 20 min for the actual test. In the FCT, participants viewed an incomplete digital poster for a fictional tourism agency and were asked to complete it creatively using a digital app. The fixed fragments—always in the foreground—were designed to evoke stereotypical responses: a circle in the top-right corner (usually seen as the sun), an ellipse in the bottom-right corner (often treated like a lake or water body), and a narrow vertical rectangle in the bottom-left corner (commonly seen as a tree). Participants could add as many basic shapes (square, circle, triangle, and an “S”-shaped figure) as desired, change their colors (including those of the fixed fragments), and insert text. The task was completed on tablets, with some participants using the device’s digital keyboard to add emojis via the text box. These elements contributed to the final creativity score, which was based on a comprehensive, judge-based evaluation of the entire drawing. Scoring employed the consensual assessment technique (Baer and Kaufman, 2019), emphasizing real-world, expert-based judgments of creativity. Five trained judges with experience in creativity assessment evaluated the entire sample. The scoring process involved multiple steps.

1) Familiarization and Gestalt Rating. The judges first assessed a secondary variable, Gestalt, which determined whether the drawing was perceived as a unified whole or as a collection of separate elements. Although this step was not included in the final analysis, it helped ensure that judges understood the full range of responses and maintained a consistent perspective.
2) Criterion Development. After reviewing the sample, judges and researchers collaboratively established scoring criteria for creativity, focusing on two main aspects: appropriateness (such as relevance to the tourism poster theme, integration of fixed fragments, visual composition, and communicative clarity) and originality (including thematic novelty, unconventional techniques, and creative reinterpretation of fixed elements). These criteria served as guiding principles for comprehensive scoring.
3) Scoring Rounds. Five judges independently rated all drawings, with each drawing receiving one rating from each judge. The scoring process was conducted in five rounds. The first three rounds, each comprising approximately 5% of the sample, served as calibration phases. During these rounds, drawings that showed substantial disagreement among judges, indicated by a standard deviation greater than one across ratings, were reviewed and discussed to resolve discrepancies and refine the scoring criteria. The final two rounds covered the remaining 85% of the sample, with periodic reliability checks conducted throughout to ensure scoring consistency.

All five judges evaluated the entire sample, allowing direct assessment of inter-rater reliability. Internal consistency among the raters’ scores was high, with Cronbach’s alpha (*α*) and McDonald’s omega (*ω*) both at 0.92. Observed scores were computed as the average of the five judges’ ratings.

#### Procedure

The study procedure is detailed in Figure 2. All measurements were conducted across three weekly sessions scheduled during regular school hours. The timing of these sessions was adjusted to align with each school’s preferences and minimize disruption, in accordance with the ethics committee’s guidelines. All sessions were carried out by professional psychologists.

**Figure 2:**
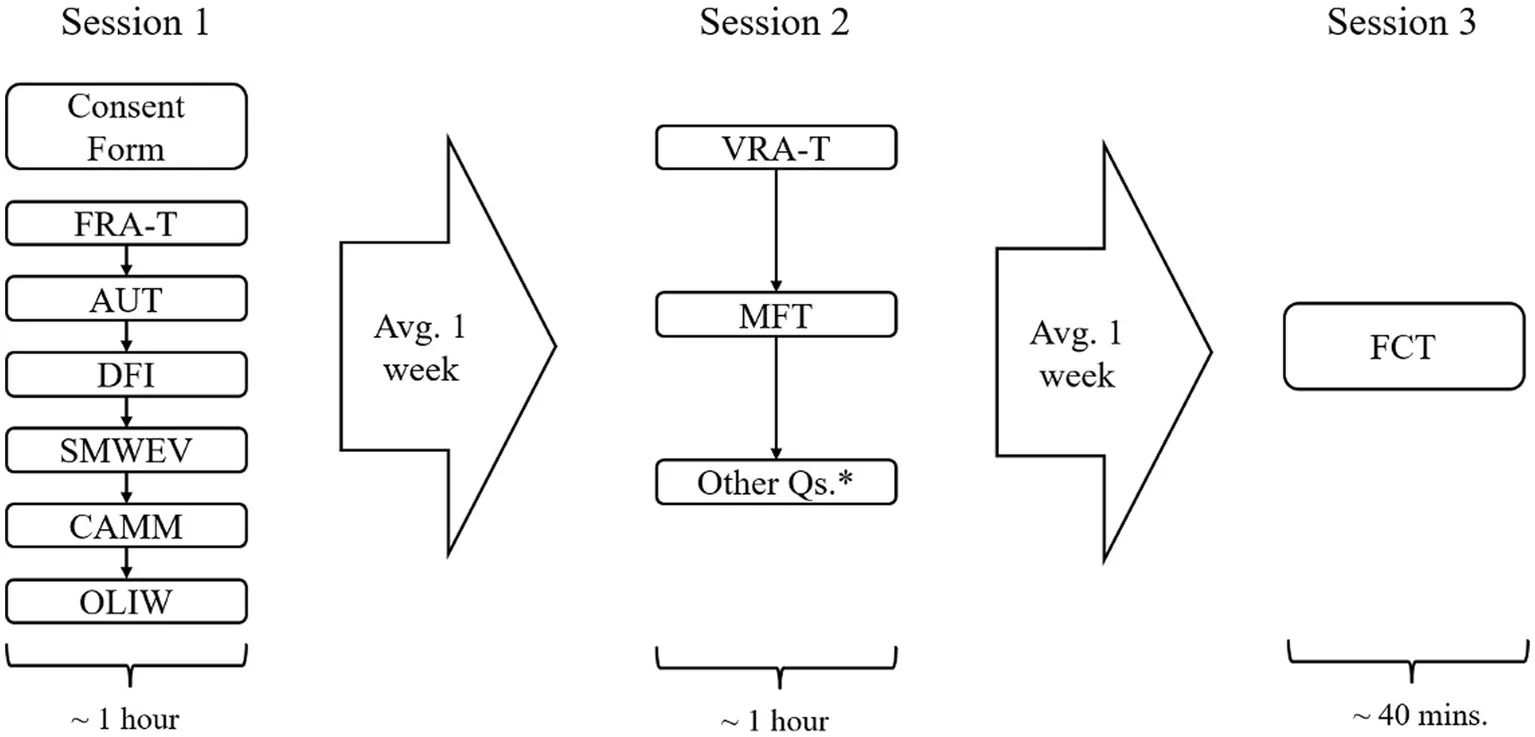
Procedure diagram. FRA-T, Figural Remote Associations Task; AUT, Alternate Uses Test; DFI, Daydreaming Frequency Inventory; SMWEV, Scale of Mind Wandering and Emotional Valence; CAMM, Child and Adolescent Mindfulness Measure; OLIW, OLIW-Youth questionnaire; VRA-T, Verbal Remote Associations Task; MFT, Mathematical Fluency Test; FCT, Figures and Colors Test. *In the second session two mental health questionnaires were administered that are not part of this publication.

During Session 1, participants completed the FRA-T followed by the AUT. The administrator read the instructions aloud for each task, answered participants’ questions, and ensured adherence to time limits. After finishing the tasks, participants had up to 20 min to individually complete self-report measures of daydreaming, mind wandering, mindfulness, and playfulness. In Session 2, participants completed the VRA-T, the MFT, and two questionnaires on psychological well-being and emotional regulation, not included in this study. As in the first session, the administrator provided guided instructions, and participants completed self-report questionnaires independently. During Session 3, participants completed the FCT with guided instructions. After completing a sampling experience study (the results of which are not reported here), each participant received a movie ticket as compensation for their time.

#### Data analysis

All analyses were performed using R (version 4.4.1; R Core Team, 2024) and Mplus (version 8.11; Muthén and Muthén, 2017), through the *Mplus Automation* package (Hallquist and Wiley, 2018). For the structural equation models (SEM), the Weighted Least Squares Mean and Variance Adjusted (WLSMV) estimator was employed (Muthén and Muthén, 2017). Given that the sample was clustered at the school level and exhibited variations in gender distribution, all structural models were estimated while controlling for both school and gender to mitigate potential confounding effects and to account for how these categorical variables influence the independent latent variables. The analysis was conducted in three steps.

1) Descriptive Analysis. Observed scores were used to calculate descriptive statistics (see Table 4) and to compute Pearson’s correlations between variables.
2) Structural Equation Modeling (SEM). A SEM was fitted to explore the predictive relationships between questionnaire-based variables and each creativity measure, while controlling for gender and school. The Weighted Least Squares Mean and Variance Adjusted (WLSMV) estimator (Muthén and Muthén, 2017) was selected because it handles ordinal and binary data and provides global fit indices. SEM allows modeling latent variables and accounts for the ordinal nature of Likert-type items and measurement error.
3) Interaction Models. Interaction effects were analyzed between the Daydreaming and Mindfulness scores, as well as between the Daydreaming and Intellectual Playfulness scores. A total of 10 models were estimated—one for each interaction across the creativity measures. Since the current version of Mplus does not support interaction terms with the WLSMV estimator, the Robust Maximum Likelihood (MLR) estimator was used instead. These models maintained the same structure as the global SEM, with fit assumed to be comparable. Due to the computational complexity of modeling interactions between latent variables derived from ordinal items, smaller models were preferred over incorporating interaction terms into the global SEM. However, testing multiple models increases the risk of Type I error. To address this, a Bonferroni-adjusted significance level of *p* ≤ 0.005 was applied, preserving an overall alpha level of 0.05 for this analysis. Although conservative, this approach provides confidence that statistically significant effects are unlikely to be due to chance.

**Table 4:** Descriptive statistics for model variables.

Variable	*N*	x̄ (s.d.)	Range	Skew	Kurtosis
Fluency	325	6.13 (2.63)	1–21	0.85	2.36
Flexibility	318	3.91 (1.37)	1–8	0.27	−0.40
Originality	318	2.43 (0.64)	1.00–4.00	−0.01	−0.65
VRA-T	391	5.34 (3.94)	0–12	0.03	−1.39
FRA-T	391	7.66 (4.26)	0–13	−0.62	−0.90
FCT	259	3.69 (1.28)	1.00–7.00	−0.09	−0.28
Math. Fluency	313	2.31 (2.17)	0–13	1.30	2.40
Mindfulness	322	2.79 (0.98)	1.00–5.00	0.15	−0.88
Daydreaming	324	3.43 (0.80)	1.42–5.00	−0.40	−0.51
Positive MW	297	2.86 (0.54)	1.00–4.00	−0.24	−0.22
Negative MW	288	2.40 (0.72)	1.00–4.00	0.04	−0.86
Other directed	329	5.60 (1.08)	1.67–7.00	−1.13	1.46
Lighthearted	326	4.31 (1.44)	1.00–7.00	−0.19	−0.67
Intellectual	295	4.76 (1.02)	1.50–7.00	−0.34	−0.05
Whimsical	299	4.92 (1.07)	2.00–7.00	−0.21	−0.42
General Playfulness	296	5.45 (1.04)	1.00–7.00	−0.86	1.26

As described before, in steps 2 and 3 of the analysis, the AUT test was treated as a single factor named “Verbal Divergent thinking.”

## Results

### Descriptive statistics and correlations

Tables 4, 5 display the descriptive statistics and pairwise correlations based on observed values.

**Table 5:** Correlation matrix between observed variables.

Variable	1	2	3	4	5	6	7	8	9	10	11	12	13	14	15
1. AUT Fluency	-														
2. AUT Flexibility	0.828*** (295)	-													
3. AUT Originality	0.678*** (318)	0.660*** (318)	-												
4. VRA-T	0.134* (320)	0.096 (313)	0.065 (313)	-											
5. FRA-T	0.241*** (320)	0.197*** (313)	0.158** (313)	0.185*** (391)	-										
6. FCT	0.152* (214)	0.097 (214)	0.125 (209)	0.298*** (253)	0.250*** (253)	-									
7. Math. Fluency	0.096 (262)	0.100 (261)	0.047 (261)	0.368*** (312)	0.198*** (312)	0.329*** (207)	-								
8. Mindfulness	−0.060 (291)	−0.086 (285)	−0.065 (285)	−0.066 (316)	−0.009 (316)	0.047 (220)	0.064 (260)	-							
9. Daydreaming	0.136* (289)	0.141* (283)	0.095 (283)	0.176** (318)	0.130* (318)	0.177** (225)	0.093 (261)	−0.380*** (315)	-						
10. Positive MW	0.033 (267)	0.043 (264)	−0.036 (264)	−0.166** (291)	0.035 (291)	−0.127 (198)	−0.075 (241)	0.087 (296)	0.069 (291)	-					
11. Negative MW	0.015 (263)	0.036 (259)	−0.007 (259)	0.135* (282)	0.037 (282)	0.061 (194)	−0.041 (233)	−0.676*** (287)	0.474*** (283)	−0.308*** (281)	-				
12. Other Directed	0.143* (294)	0.066 (288)	0.026 (288)	0.017 (322)	0.107 (322)	−0.045 (227)	−0.003 (265)	−0.101 (319)	0.050 (321)	0.323*** (294)	−0.051 (286)	-			
13. Lighthearted	−0.033 (292)	−0.048 (286)	−0.077 (286)	−0.179** (319)	−0.051 (319)	−0.102 (225)	−0.160** (262)	0.246*** (317)	−0.099 (318)	0.308*** (292)	−0.371*** (284)	0.195*** (326)	-		
14. Intellectual	0.244*** (266)	0.193** (260)	0.191** (260)	0.017 (289)	−0.065 (289)	0.080 (203)	0.056 (240)	0.019 (288)	0.122* (289)	0.412*** (269)	−0.181** (262)	0.340*** (295)	0.261*** (293)	-	
15. Whimsical	0.212*** (269)	0.182** (264)	0.105 (264)	0.033 (293)	−0.020 (293)	0.039 (206)	0.079 (242)	−0.042 (292)	0.299*** (291)	0.325*** (272)	0.025 (264)	0.259*** (296)	0.222*** (294)	0.454*** (287)	-
16. General Playfulness	0.067 (266)	−0.010 (260)	−0.032 (260)	−0.077 (290)	−0.002 (290)	−0.109 (201)	0.006 (241)	0.006 (289)	−0.015 (288)	0.444*** (269)	−0.234*** (260)	0.529*** (293)	0.347*** (291)	0.516*** (285)	0.461*** (290)

Pairwise N in parenthesis. *p < 0.05, **p < 0.01, ***p < 0.001.

Following the Gignac and Szodorai’s (2016) guidelines for individual difference researchers, who suggested considering correlations of 0.10, 0.20, and 0.30 as relatively small, typical, and relatively large, respectively, a large correlation was found between AUT fluency and AUT originality scores (*r* = 0.678, *p* < 0.001), indicating that participants who generated more ideas also tended to produce more original ones. There was a stronger correlation between AUT fluency and AUT flexibility scores (*r* = 0.828, *p* < 0.001), as expected, since the flexibility score depends to some extent on the fluency score. A less-than-typical but significant correlation was observed between the figural and verbal remote association tasks (VRA-T and FRA-T, *r* = 0.185, *p* < 0.001). The correlation between figural creativity (FCT) and the verbal remote association task was slightly larger than that between the FCT and the figural one (*r* = 0.298 with VRA-T, and 0.250 with FRA-T). Daydreaming showed small positive correlations with AUT Fluency (*r* = 0.136, *p* < 0.05), AUT Flexibility (*r* = 0.141, *p* < 0.05), VRA-T (*r* = 0.176, *p* < 0.01), FRA-T (*r* = 0.130, *p* < 0.05), and FCT (*r* = 0.177, *p* < 0.01). Mindfulness had large negative correlations with Daydreaming (*r* = −0.380, *p* < 0.001) and Negative MW (*r* = −0.676, *p* < 0.001). Within the domain of playfulness, all scales had large correlations with Positive MW. Negative MW showed large, small, and typical negative correlations with Lighthearted, Intellectual, and General playfulness, respectively. Mindfulness showed a typical correlation with Lightheartedness, and Frequency of Daydreaming displayed a large correlation with Whimsical and a small correlation with Intellectual.

### Global structural equation model

The structural equation model examined how psychological and dispositional variables predict five creativity outcomes: Verbal Divergent Thinking, FRA-T, VRA-T, FCT, and MFT. Verbal Divergent Thinking was significantly and positively predicted by Intellectual Playfulness (*β* = 0.37, *p* = 0.001). Notably, General Playfulness (*β* = −0.36, *p* = 0.019), Negative MW (*β* = −0.30, *p* = 0.045), and Positive MW (*β* = −0.25, *p* = 0.012) were negatively associated with Verbal Divergent Thinking. FRA-T performance was positively predicted by Daydreaming (*β* = 0.22, *p* = 0.005) and Other-directed playfulness (*β* = 0.27, *p* = 0.035). The VRA-T was positively predicted by Daydreaming (*β* = 0.20, *p* = 0.014) and negatively predicted by Positive MW (*β* = −0.19, *p* = 0.035). The FCT was positively predicted by the CAMM (*β* = 0.38, *p* = 0.019) and Daydreaming (*β* = 0.19, *p* = 0.049). The MFT was positively predicted by Daydreaming (*β* = 0.43, *p* = 0.015), while Lighthearted playfulness showed a negative effect (*β* = −0.57, *p* = 0.002).

Gender differences in the independent variables were also considered in the model. Female participants showed positive associations with the Daydreaming score (*r* = 0.12, *p* < 0.001) and Negative MW (*r* = 0.14, *p* < 0.001), and negative associations with the CAMM (*r* = −0.16, *p* < 0.001) and Lighthearted playfulness (*r* = −0.17, *p* < 0.001).

The model demonstrated acceptable fit across multiple indices: Comparative Fit Index (CFI) = 0.936, Tucker-Lewis Index (TLI) = 0.933, Root Mean Square Error of Approximation (RMSEA) = 0.024 with a 90% confidence interval of [0.022, 0.026], and Standardized Root Mean Square Residual (SRMR) = 0.073. The only evidence of misfit was the chi-squared statistic, χ^2^(5538) = 6832.81, *p* < 0.001. Overall, these values suggest that the model adequately captures the relationships among the latent variables, accounts for measurement error, and preserves the ordinal nature of the questionnaire data.

Table 6 presents the model results, while Figure 3 provides a simplified visual representation of the fitted model.

**Table 6:** Structural equation model for verbal divergent thinking, convergent thinking and creativity.

Variable	Verbal DVT			FRA-T			VRA-T			FCT			MFT		
	Est	S.E	*p*	Est	S.E	*p*	Est	S.E	*p*	Est	S.E	*p*	Est	S.E	*p*
Mindfulness	−0.16	0.13	0.231	0.14	0.12	0.257	0.13	0.13	0.330	**0.38**	**0.16**	**0.019**	0.25	0.28	0.387
Daydreaming	0.06	0.08	0.461	**0.22**	**0.08**	**0.005**	**0.20**	**0.08**	**0.014**	**0.19**	**0.10**	**0.049**	**0.43**	**0.18**	**0.015**
Positive MW	**−0.25**	**0.10**	**0.012**	0.09	0.10	0.411	**−0.19**	**0.09**	**0.035**	−0.16	0.11	0.134	−0.35	0.19	0.065
Negative MW	**−0.30**	**0.15**	**0.045**	−0.04	0.15	0.794	−0.02	0.15	0.897	0.18	0.19	0.339	−0.46	0.32	0.150
Other directed	0.21	0.13	0.108	**0.27**	**0.13**	**0.035**	0.17	0.12	0.133	−0.04	0.15	0.801	0.09	0.22	0.673
Lighthearted	−0.10	0.09	0.267	−0.04	0.08	0.668	−0.16	0.08	0.058	−0.03	0.10	0.795	**−0.57**	**0.19**	**0.002**
Intellectual	**0.37**	**0.12**	**0.001**	−0.17	0.12	0.165	0.07	0.12	0.575	0.25	0.13	0.061	0.04	0.30	0.887
Whimsical	0.20	0.12	0.084	−0.02	0.11	0.856	0.15	0.11	0.150	0.04	0.12	0.755	0.30	0.25	0.235
General playfulness	**−0.36**	**0.15**	**0.019**	−0.09	0.15	0.556	−0.20	0.13	0.143	−0.12	0.17	0.504	0.08	0.28	0.775
Gender (F)	0.18	0.15	0.235	0.09	0.15	0.567	−0.01	0.15	0.969	0.31	0.16	0.058	−0.07	0.32	0.817
School B	0.26	0.30	0.375	−0.44	0.46	0.342	−0.67	0.59	0.255	−0.31	0.44	0.482	−0.87	0.93	0.352
School C	−0.02	0.16	0.898	−0.39	0.22	0.083	−0.54	0.30	0.066	−0.35	0.20	0.077	−0.70	0.52	0.176

Verbal DVT, Verbal Divergent Thinking; FRA-T, Figural Remote Association Test; VRA-T, Verbal Remote Association Test; FCT, Figures and Colors Test; Positive MW, Positive Mind Wandering; Negative MW, Negative Mind Wandering; MFT, Mathematical Fluency Test. This analysis considered all 405 participants. Bold values represent statistically significant estimates (*p* < 0.05).

**Figure 3:**
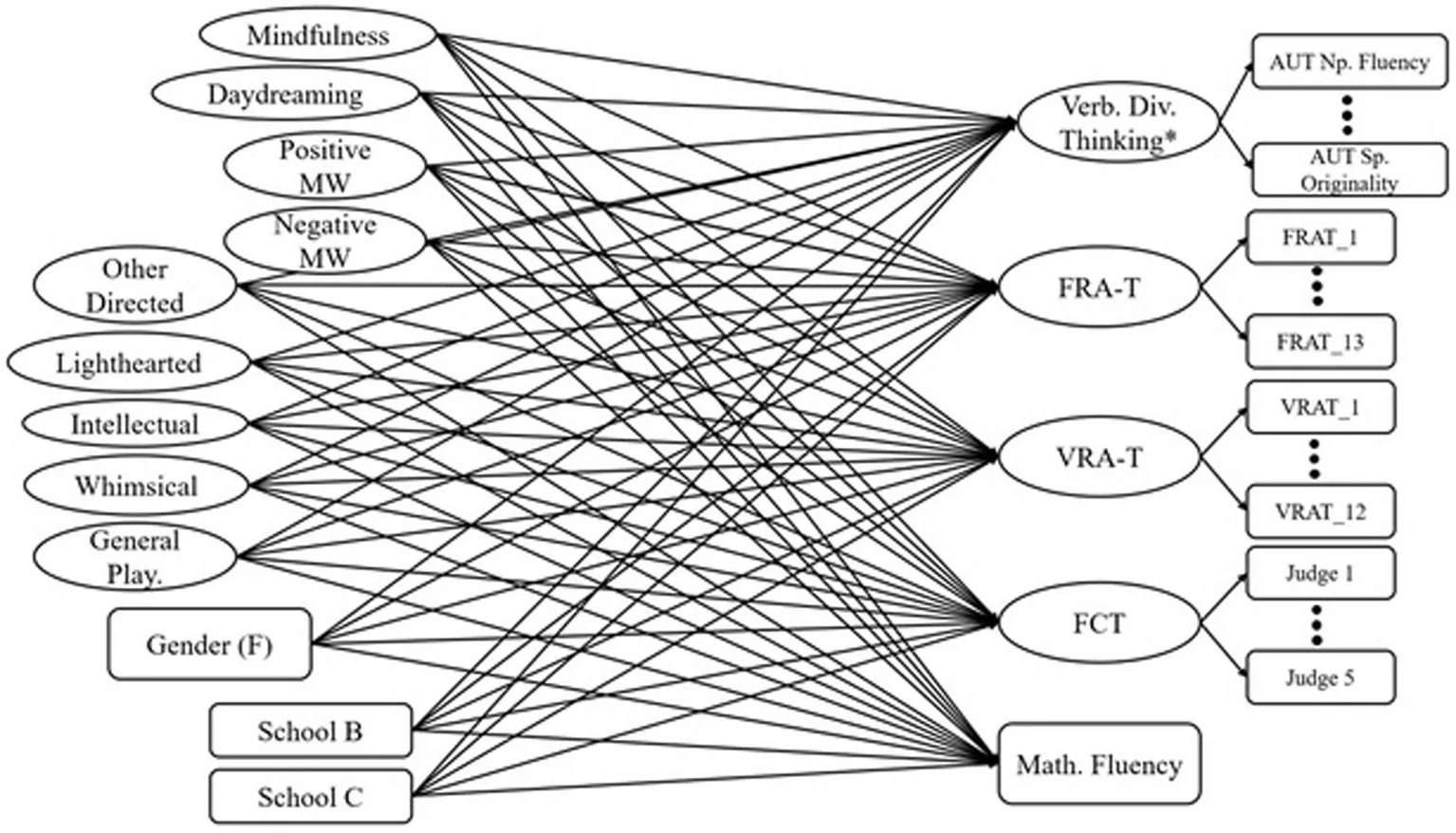
Simplified diagram of the structural equation model. Note: Latent variables are represented by ellipses and observed variables by rectangles. For clarity, the items composing the independent variables (shown on the right) are omitted, as are the lines representing correlations among independent and dependent variables. *Not all AUT scores are displayed in the figure; all six scores (fluency, flexibility, and originality for each item) load directly onto Verbal Divergent Thinking.

*Interaction Models*. To explore potential moderation effects, 10 interaction models were tested to examine whether Daydreaming interacted with either CAMM or Intellectual playfulness in predicting creativity outcomes. As described in the data analysis sub-section, a Bonferroni-adjusted significance threshold of *p* ≤ 0.005 was applied to account for multiple comparisons. These models included 358 to 394 participants. Of the ten models, only one interaction reached statistical significance: the interaction between daydreaming and mindfulness in predicting FCT scores. This model showed improved explanatory power, with R^2^ increasing from 0.140 to 0.207 relative to the same model without the interaction effect, indicating that the interaction accounted for an additional 6.7% of the variance in FCT scores, a 47.9% increase compared to the base model. The interaction term was negative (*β* = −0.23, SE = 0.07, *p* = 0.001), suggesting that the effect of one variable diminishes as the other increases. Details of this model are presented in Table 7.

**Table 7:** Results for the interaction model for FCT by DD*CAMM.

Effect	Step 1			Step 2		
	Estimate	S.E.	*p*	Estimate	S.E.	*p*
Daydreaming	0.27	0.08	0.001	0.29	0.07	<0.001
Mindfulness	0.20	0.08	0.010	0.23	0.08	0.003
Mindfulness * Daydreaming				−0.23	0.07	0.001
Gender (F)	0.17	0.14	0.225	0.13	0.14	0.345
School B	−0.47	0.25	0.061	−0.49	0.24	0.046
School C	−0.46	0.13	<0.001	−0.45	0.13	<0.001
R^2^			0.140			0.207
Δ R^2^						0.067

Overall *n* = 358.

To aid interpretation, Figure 4 shows that higher mindfulness predicts higher figural creativity when daydreaming is low. However, at high levels of daydreaming, the relationship reverses: higher mindfulness is associated with lower creativity. This pattern reflects a moderating interaction rather than a simple main effect. The mindfulness values plotted correspond to the minimum and maximum estimated values of the latent variable in the sample, ensuring the visualization accurately represents the empirical range of data.

**Figure 4:**
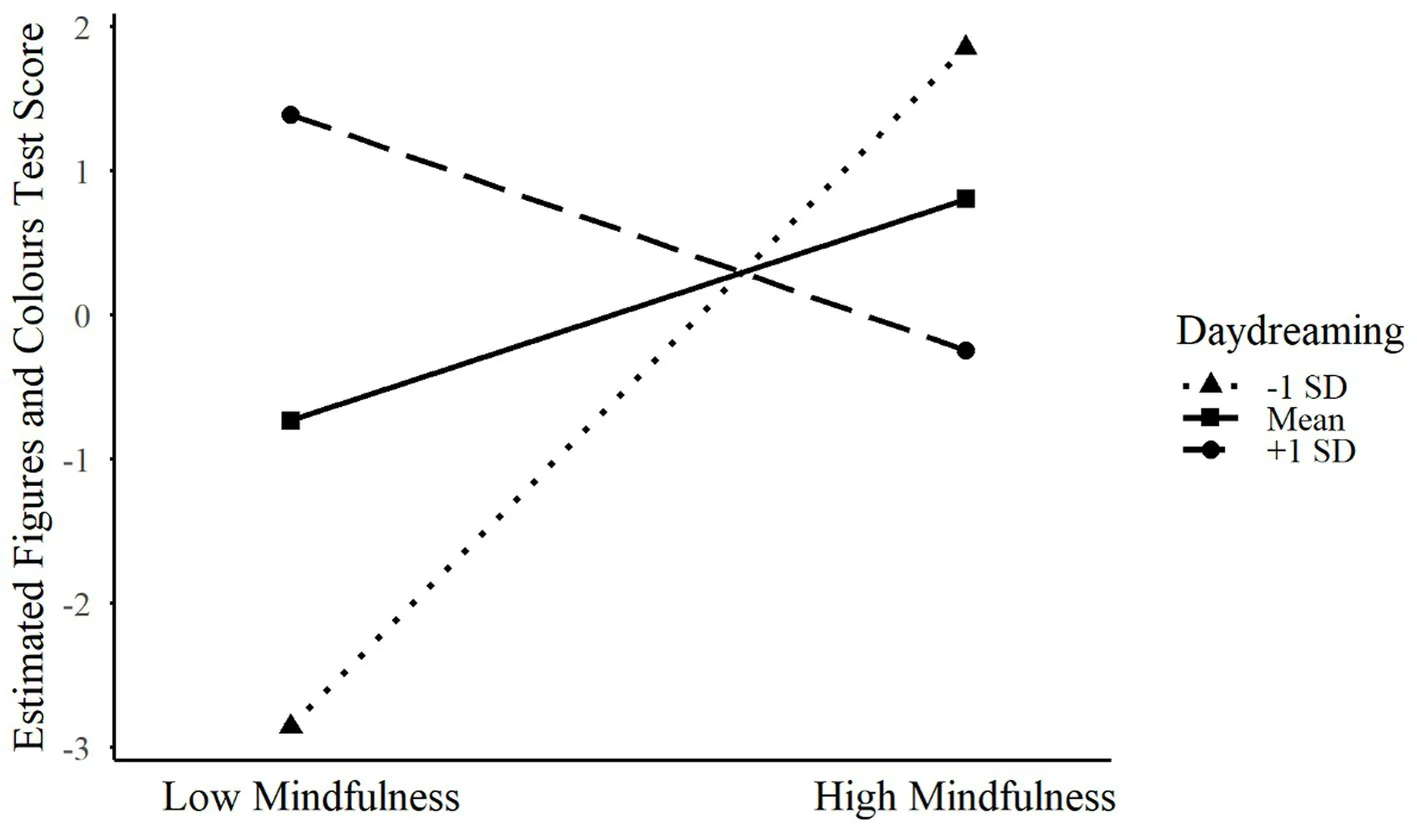
Interaction plot for daydreaming with mindfulness on FCT score.

## Discussion

This study examined how daydreaming frequency, the emotional tone of mind wandering, mindfulness, and multiple dimensions of playfulness relate to creativity across verbal, figural, and mathematical creative tasks in adolescence. Inspection of the correlations showed that Daydreaming had small positive correlations with five out of the seven creativity scores. Mindfulness was negatively related to Daydreaming, consistent with previous research suggesting that mindfulness reduces unintentional and negatively biased mind wandering (Mrazek et al., 2012; Mrazek et al., 2014b; Torres-Irribarra et al., 2019; Zedelius and Schooler, 2015). Correlations also indicated that a playful disposition is connected to a tendency to engage in constructive, imaginative daydreaming. The SEM results indicated that, after accounting for gender, schools and all dispositional factors, the relationships between daydreaming and four creativity tests were significant; however, its association with the verbal divergent thinking factor was not significant. These findings support the idea that a tendency to engage in spontaneous, internally generated thoughts is related to the cognitive processes underlying these creativity tests. Furthermore, these results suggest that daydreaming can act as a cognitive resource rather than a distraction, especially in tasks that rely on associative thinking (Rodriguez-Boerwinkle et al., 2025). Contrary to expectations, positive mind wandering was negatively associated with both verbal creativity measures.

The mindfulness measure used in this study showed no consistent associations with creativity across domains. In the SEM, mindfulness predicted creativity only in the FCT, the only task in our battery that involved idea evaluation, integration, and refinement to produce a coherent outcome. This indicates that, at least in this age group, a disposition toward mindful awareness may be essential for complex creative tasks that involve evaluation or revision, highlighting how controlled or regulated processes affect creative performance in activities that, beyond mere divergent or associative thinking, require the creation of a fully finalized product (Preiss, 2022b; Rodriguez-Boerwinkle et al., 2025; Zedelius and Schooler, 2015).

We expected to find systematic relationships between our creative measures and Intellectual playfulness, but this variable was significantly related only to the Verbal Divergent Thinking Factor in the SEM. The other playfulness sub-scales had varied effects in the SEM: General playfulness was negatively associated with Verbal Divergent Thinking, Other-directed playfulness predicted the FRA-T positively, and Lighthearted playfulness predicted mathematical fluency negatively. Thus, the results do not support the idea that playfulness, as measured in this study, is associated with creativity across domains in a consistent way. Also, these findings suggest that a broad “playfulness” construct might obscure more specific and sometimes opposing influences on creative performance.

It is worth noting that there were no gender or school differences in the creativity measures when the trait measures were considered.

Next, we discuss these results in light of the hypotheses presented above regarding the relationship between mind wandering and creativity. Our results suggest that the idea that mind wandering may serve as a developmental scaffold in adolescence is worth exploring (Rodriguez-Boerwinkle et al., 2025). Daydreaming frequency was consistently associated with most of our creativity measures. Additionally, the association between daydreaming and performance on tasks requiring associative activation and loose semantic search—such as the VRA-T and FRA-T—provides indirect support for the hypothesis that mind wandering facilitates access to distant associations (Baird et al., 2012; Irving et al., 2024). This mechanism might be especially relevant for tasks that rely on retrieving or combining related concepts. Beyond these two measures, in the SEM model, daydreaming was also linked to tasks involving divergent thinking in mathematics and the creation of complex figures, suggesting that this disposition may play an important role across diverse creative task demands (Preiss, 2022b). Yet, as previously noted, the interaction between mindfulness and daydreaming in predicting FCT performance showed that higher mindfulness predicts higher figural creativity when daydreaming is low, but the relationship reverses at high levels of daydreaming. In complex creative tasks that involve both idea generation and evaluation, a positive association between creativity and mindfulness might shift to negative at higher levels of daydreaming. These results underscore the importance of examining the interplay of attentional dispositions, suggesting that the role of mindfulness in creativity may be conditional on broader cognitive contexts.

Beyond these hypothesis, it is worth noting that positive MW was negatively associated with the verbal remote association measure and both forms of emotionally laden mind wandering were negatively associated with verbal divergent thinking, suggesting that spontaneous thought with affective content may hinder performance in these verbal creativity measures, even in the case of positive emotions, a finding that goes against other evidence showing that positive mood, especially a happy one, enhances creativity (Baas, 2019; Baas et al., 2008). Still, neither scale was associated with any of the other three creative tests in the mathematical or figural domain.

Playfulness effects were limited, and when present, they seemed to depend on the cognitive demands of each task. As noted, Intellectual playfulness was positively associated with verbal divergent thinking, providing indirect support for the idea that playful exploration of ideas, consistent with intentionally adjusting spontaneous thought, can boost creative output. In contrast, general playfulness was negatively associated with the same measure, challenging the belief that playfulness always promotes creativity; moreover, this dimension showed no connection to any other creative tasks. Lighthearted playfulness was negatively associated with mathematical divergent thinking, which relies on disciplined, rule-based recombination rather than free-form thinking. The positive link between Other-directed playfulness and FRA-T performance is harder to interpret, since these tasks did not involve a social component. Overall, these varied effects suggest that future research on playfulness and creativity should focus on the role of intentionality and cognitive guidance in creative processes, which are more proper of Intellectual playfulness, rather than assuming a universal, undifferentiated effect of playfulness.

### Limitations

Despite its contributions, this study has several limitations. First, the sample consisted solely of Chilean high school students, which may limit the applicability of the findings to other age groups or cultural contexts. Second, the cross-sectional design prevents making causal claims about the relationships among attentional dispositions, playfulness, and creativity. Third, although the study used multiple creativity measures, attentional dispositions were assessed through self-report, which can be biased. Fourth, the interpretation of our results must consider that some of the measures used are exploratory, especially our figural and mathematical measures, and that others involve translation into a context different from the one in which they originated. The properties of these measures still need further research to be completely validated. Finally, the interaction models were constrained by computational constraints, requiring simplified structures that might not fully capture the complexity of the relationships among latent variables. Future research should address these limitations by using more diverse and larger samples and longitudinal designs.

### Practical implications

The study’s findings highlight several key implications for high school education and fostering creativity. First, recognizing the positive role of daydreaming in creativity, educators and curriculum developers should create space for spontaneous thought, especially during tasks involving idea generation (Preiss, 2022b; Zedelius et al., 2021). Because daydreaming is associated with higher creativity across domains, schools should provide students with opportunities that encourage spontaneous thinking (Preiss and Carmona, 2023) and teach them how to regulate their attention at different stages of creative problem-solving. Second, because mindfulness showed benefits only on tasks requiring evaluation, it should be used strategically rather than in all situations, particularly in contexts that involve analytical strategies (Zedelius and Schooler, 2015) and the creation of finalized products. Third, the contrasting effects of different dimensions of playfulness underscore the need to use play strategically in instruction, tailoring game-based interventions to specific task requirements and pedagogical goals. Overall, the results suggest that fostering creativity in adolescence involves balancing structure with openness in instruction and supporting students’ development of metacognitive awareness of the role that attentional processes play in creativity and learning.

In conclusion, this research highlights the importance of exploring the links between attentional dispositions, playfulness, and creativity across multiple facets and domains of creativity. Future research should use longitudinal approaches to understand how these relationships develop during childhood and adolescence, especially the conditions where spontaneous thought is helpful, neutral, or harmful. Clarifying how adolescents balance spontaneous and controlled thought through personal awareness and self-regulation may help educators create environments that foster creative potential while supporting emotional and attentional growth.

## Data Availability

The datasets presented in this study can be found in online repositories. The names of the repository/repositories and accession number(s) can be found at: https://osf.io/ng7zx/files/osfstorage.
